# Gender Differences in HIV, HCV risk and Prevention Needs Among People who Inject drug in Vietnam

**DOI:** 10.1007/s10461-022-03932-x

**Published:** 2022-11-28

**Authors:** Hoang Thi Giang, Nguyen Quang Duc, Pham Minh Khue, Catherine Quillet, Khuat Thi Hai Oanh, Nham Thi Tuyet Thanh, Roselyne Vallo, Jonathan Feelemyer, Vu Hai Vinh, Delphine Rapoud, Laurent Michel, Didier Laureillard, Jean Pierre Moles, Don Des Jarlais, Nicolas Nagot, Duong Thi Huong

**Affiliations:** 1 Faculty of Public Health, Haiphong University of Medicine and Pharmacy, 72A, Nguyen Binh Khiem, Ngo Quyen district, Haiphong city, Vietnam; 2 Pathogenesis and control of chronic & emerging infections, Etablissement Français du Sang, University of Montpellier, INSERM, University of Antilles, Montpellier, France; 3 Supporting Community Development Initiatives, Hanoi, Vietnam; 4 New-York University, NewYork, NY, USA; 5 Dept of Infectious and Tropical Diseases, Viet Tiep Hospital, Hai Phong, Vietnam; 6 Pierre Nicole Center, CESP/Inserm 1018, French Red Cross, Paris, France; 7 Infectious Diseases Department, Caremeau University Hospital, Nimes, France

**Keywords:** Women, People who inject drugs, HIV risk, Prevention needs, Vietnam

## Abstract

We examined gender differences among people who inject drug (PWID) in Hai Phong, Vietnam in term of blood-borne infections, risk behaviors, and access to care. Using respondent-driven-sampling surveys, we recruited 3146 PWID from 2016 to 2018. Inclusion criteria included a positive urine test for heroin and recent injection marks. There were 155 female PWID (4,9%), including 82 at RDS-2016, 32 at RDS-2017 and 38 at RDS-2018. The age mean was 36.3 ± 7.2 years. The majority of female PWID had less than high school education (90.9%) and were unemployed (51.3%). There was no difference in the proportion of HIV and HCV positive by gender. However, women had several significant differences in risk behaviors than men in multivariable logistic regression. Being a woman was independently associated with being unemployed, being a sex worker, having unstable housing, having uses drugs for less than 5 years, more use of methamphetamine, having a partner who ever injected drugs, and less access to methadone treatment. Interventions targeting female PWID are needed, possibly through community organizations and peer educators.

## Introduction

Globally, the number of people who inject drugs (PWID) is approximately 11 million, of whom 1.4 million are living with HIV and 5.6 million with hepatitis C (HCV) according to World Drugs Report - UNODC 2019 [1]. No global population size estimates of females who inject drugs (FWID) are available, and data gaps exist in nearly all countries. According to recent estimates, FWID range from 10% to over 30% in some parts of Europe; approximately 20% in Eastern Europe, Central Asia and Latin America; 10–20% in parts of Africa; 20% in China and Viet Nam, and at least 10% in other parts of Asia, corresponding to approximately 3.5 million FWID globally [2, 3]. A previous study has shown variation in the female: male odds ratios for HIV prevalence but overall there was a modest but significantly higher HIV prevalence among females with an overall odds ratio of 1.18. FWID also had higher rates of infection compared to male who inject drugs (MWID) in Eastern Europe (33.0% vs. 27.9%), Western Europe (42.8% vs. 40.3%), Latin America (38.5% vs. 34.6%) and North America (34.5% vs. 31.3%) [2].

In addition to the risk of HIV and HCV exposure, FWID face multilevel drivers that increase their vulnerabilities to sexually transmitted diseases, impaired physical and mental health [4], particularly among female sex workers. FWID experience stigma and discrimination, including from within drug user networks, due to gendered social norms and the role of women as primary care givers. This may affect access to treatment and prevention services, and may also lead to higher levels of sexual risk exposures [2, 5, 6]. In many settings, sex work is criminalized, with legal sanctions directed towards women who engage in sex work rather than their clients, who are predominantly men [7]. Violence, or the threat of violence, is also a significant contributor to HIV and HCV risk behaviors among PWID, serving to undermine women’s ability to practice safe sex and safer drug use with intimate partners [8] and during sex work [7]. In some regions of Africa, women who have experienced physical or sexual intimate partner violence are 1.5 times more likely to acquire HIV than women who have not experienced such violence [9]. The results from a nationwide survey among Iranian female sex workers showed that female sex workers with high levels of sexual activity use more drugs than those with less activity. The main reason was that they know drug use is an effective factor in reducing depression or obsessive-compulsive disorder, and know that drugs can help eliminate sexual reluctance, increase emotional tendencies, increase pleasure in sexual relationships, and prevent sexual disputes. Hence, those that use drugs are able to have more customers every day and earn more money [10].

In Vietnam, there are an estimated 189,000 PWID and HIV prevalence among PWID was 12.7% in 2020 [11], with large variations across regions. Injecting drug use remains the leading HIV transmission mode in Vietnam, although the rate of transmission among MSM is approaching parity [11]. Modelling in 2018 estimated that 25% of new infections occurred among PWID including 37% among women at low risk and 2% among female sex workers [12]. Mortality rates among PWID remain high in Vietnam despite very successful HIV and methadone programs. Crude mortality rates are approximately 4.3 (95% Confidence interval (CI): 3.3–5.4) per 100 person-years of follow-up (PYFU) among HIV-positive PWID and 1.9 (CI: 1.4–2.6) per 100 PYFU among HIV-negative PWID. The main causes of death are tuberculosis and HIV-related diseases in the HIV-positive group, while the main causes of death are liver-related diseases and overdose in the HIV-negative group [13].

PWID also report high levels of stigma from their communities, which are often amplified in the presence of HIV infection and make it difficult for PWID to earn money, which in many cases places the burden of care on their families [14]. Beyond physical morbidity and mortality, drug use carries a significant mental health burden; 25.5% had at least one psychiatric disorder and 10.1% had 2 or more psychiatric disorders after 12 month’s follow-up in Haiphong in 2016 and FWID were more likely than males to have at least one psychiatric disorder, a major depressive disorder, or an anxiety disorder [15]. Nevertheless, data on the health risks of women who use and inject drugs are still scarce. The lack of data regarding risks among FWID, including female sex workers, transgender women, racial and ethnic minority women, and young women may affect the efficiency of HIV control efforts.

Haiphong city is one of the largest cities in Vietnam with approximately 2 million inhabitants and an estimated 10,000 PWID. The focused resources on addressing issues related to PWID such as antiretroviral treatment (ART), syringe-needle-exchange programs (SNEP) and medication for opioid use disorder (MOUD) have existed in this city for nearly 20 years [16-20]. In 2014, we conducted a respondent driven sampling survey (RDSS) among heroin injectors in Haiphong to assess the efficiency of these prevention programs and estimate the incidences of HIV and HCV infections. We reported a low HIV incidence (between 0 and 1.8 per 100 person-years (PY), contrasting with a very high HCV incidence (19.4/100 PY, 95% CI: 11.5–30.7) [21]. This paper aimed to assess the additional HIV and HCV risk, as well as the prevention and treatment needs of FWID in comparison to MWID in Haiphong city, Vietnam.

## Methods

### Study Design

We conducted a secondary analysis of data collected from 3 successive annual RDSS conducted in Hai Phong, Vietnam from 2016 to 2018 as part of the interventional Drugs & Infections in ViEtnam (DRIVE) research project. A case – control analysis was set with 1 FWID for 4 MWID. All the different FWID enrolled in the RDSS were selected as a “case group”. Only data at first enrolment were considered if an FWID was recaptured during a subsequent RDSS. For the “control group”, four MWID were randomly selected matching on age and RDSS date equivalent to each FWID case.

### Setting

The RDSS was implemented in Haiphong city with active involvement of seven community-based organization (CBO)s in several aspects of the study. The RDSS survey took place between October and January every year in two CBO offices (Light House and Friendship Arms).

### Study Population and Study Conduct

To construct the RDSS sample, twenty non-randomly identified “seeds” were recruited by CBO members for each RDSS. These included PWID with short and long histories of injecting, sex workers who inject drugs, and men who have sex with men who inject drugs from various districts. They participated in all procedures and were then instructed on how to recruit new study participants using the RDSS coupons from their injecting drug user’s networks. Each coupon was valid for one week. The proportion of coupons that were returned for inclusion in the RDSS was approximately 30% for each RDSS and the proportion of seeds that generated 3 or more rounds of responses was approximately 80% on average for the 3 RDSS. The network size of each participant was collected in RDSS Coupon Manager^®^ 3.0 software. The RDSS eligible criteria included:

- Being 18 years old and older,
- Living in Haiphong, Vietnam during the study period.
- Urine positive test for heroin and/or methamphetamine and recent injection marks on the skin examined by CBO members.

All eligible subjects who provided informed consent completed a questionnaire in Vietnamese, participating in a drug urinalysis test and a blood collection for HIV and HCV serology and for plasma banking by following an anonymous procedure at two community study sites located in urban Haiphong city. The interviewer administered a questionnaire on sexual behaviors, drug use and health care utilization. Participants were also screened for depression and anxiety using the PHQ4-scale (The Patient Health Questionnaire 4) [22]. All participants received $7.50 for the interview, $2.50 for transportation fees and $2.50 for each peer they successfully recruited. They also received an additional $2.50 to return to the study site to obtain their HIV and HCV test results one week after the RDSS. Subjects who screened HIV-positive were immediately referred to antiretroviral care. Participants were also referred to methadone treatment upon request.

### HIV and HCV Testing

HIV antibody and HCV antibody testing were conducted by the well-trained nurses of Haiphong Provincial AIDS Control Centre (PAC) at the study site using SD Bioline HIV1/2 3.0 rapid test and SD Bioline HCV rapid test (Standard Diagnostics Inc., Gyeonggi-do, Republic of Korea). HIV confirmation tests were done at PAC according to the National guidelines using Determine^™^ HIV-1/2 (Alere^™^, Waltham, USA) plus the VIKIA^®^ HIV1/2 test (Marcy-l’Etoile, Lyon, France) [23]. All the test results were returned to participants within 8 days after recruitment date.

### Cascade of HIV care

The HIV viral loads were quantitated using the COBAS HIV-1 test (Roche) in a reference laboratory. To identify PWID with ARV treatment failure, the plasma lamivudine (3TC), present in all local ART regimens, was tested by HPLC at Ho Chi Minh Pasteur Institution for all PWID with a HIV viral load of more than 1000 copies/mL.

### Data Analysis

Data were analyzed using STATA software (version 15.1, Stata Corp, College Station, Texas, USA). Chi-square tests or t-tests were used to compare demographic and behavioral characteristics by gender. Bivariate and multivariable logistic regression analysis were performed to explore the factors associated with gender. All variables with a p-value below 0.2 in the bivariate analysis were included in the multivariable analysis and the goodness-of-fit test was used to consider the suitability of the model by using McFadden’s Pseudo R2 indicator [24]. A probability level of 0.05 or less was considered statistically significant.

We defined unsafe sex when a condom was not always used (i) during sexual intercourse with a primary partner with unknown or positive HIV status and (ii) during sexual intercourse with a casual partner. A cut-point of 6 was used to identify participants with moderate/severe anxiety/depressive symptoms with the PHQ 4 [22].

## Results

### Overall Female PWID Characteristics

**Fig. 1:**
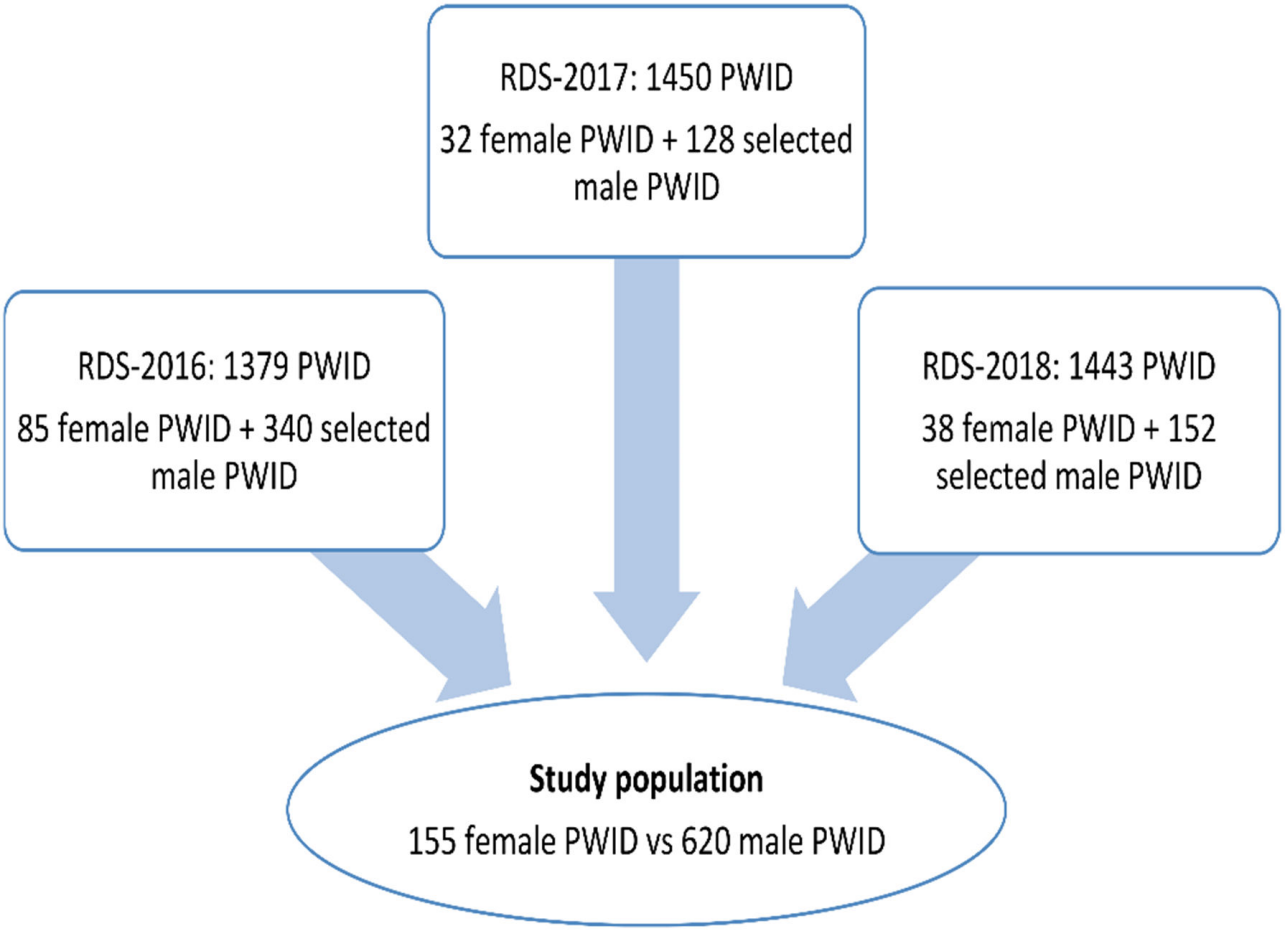
Participants’ flow chart

Of the 3146 PWID enrolled in the study over the 3 RDSS, 155 were FWID (4.9%), including 85 at RDSS-2016, 32 at RDS-2017 and 38 at RDSS-2018. Their mean age was 36.3 ± 7.2 years (Fig. 1). The selection of MWID fulfilled the initial criteria with a mean age of 36.8 ± 7.1 years (Table 1). Overall, the majority of FWID had less than high school education (90.9%) and were unemployed (51.3%). Eighty-six FWID (55.5%) had a monthly income of more than $250. Only 61 FWID had a health insurance card (39.4%). Fifteen (9.7%) had unstable housing and 10 had experienced overdose to the point of unconsciousness (6.5%) in the last 6 months. 30% of the participants were confirmed HIV-positive, and 67.1% were HCV positive.

### Socio-demographic Characteristics by Gender

As shown in Table 1, FWID were significantly more unemployed than MWID (51.3% vs. 31.3%, Chi-square = 21.04, p<0.001) and reported higher levels of unstable housing in the last 6 months compared to MWID (9.7% vs. 3.7%, Chi-square = 9.47, p < 0.01). FWID were more likely to have less than a high school education compared to MWID (90.9% vs. 73.2%, Chi-square = 21.89, p < 0.001). The percentage of FWID with an average monthly income of over $250 was slightly higher than that of MWID (50.3% vs. 40.8%, Chi-square = 4.59, p<0.05). There was no difference between FWID and MWID with respect to having a health insurance card, having an identity card (Table 1) and reporting a history of being incarcerated overnight.

### Comparison of HIV/HCV Status and Associated risk Factors by Gender

There was no difference in the prevalence of HIV and HCV by gender (FWID vs. MWID: 30.3% vs. 28.1% and 67.1% vs. 69.8%, respectively). Similarly, there was no difference in the primarily injected drug by gender (~ 100% were injecting heroin). However, with respect to drug use behavior, FWID were more likely than MWID to report injecting for less than 5 years (38.1% vs. 26.1%, Chi-square = 8.67, p < 0.01). The number of heroin injections per day among FWID were significantly higher than MWID (2.5 ± 1.0 vs. 2.2 ± 0.9, t-test = −3.22, p < 0.001). FWID were more likely to have a positive urine test for methamphetamine compared to MWID (45.8% vs. 36.3%, Chi-square = 4.76, p < 0.05), while no difference in self-reported methamphetamine use data was found. There were no differences in needle/syringes/water sharing between MWID and FWID, nor in the number of days injecting per month, in multi-drug use, in overdose and in non-injecting drug use.

FWID were more likely than MWID to report unsafe sex in the last six months (28.4% vs. 12.1%, Chi-square = 25.32, p < 0.001) and having a sex partner who ever injected drugs (38.1% vs. 2.3%, Chi-square = 186.33, p < 0.001).

### Comparison of Psychosocial Characteristics by Gender

With respect to depressive/anxiety symptoms in the last 2 weeks, FWID were more likely than MWID to have a thought of harming oneself (17.4% vs. 9.7%, Chi-square = 7.46, p < 0.05), while no differences were found for moderate/severe depressive/anxiety symptoms with the PHQ-4 (Table 1).

### Comparison of Access to care by Gender

**Fig. 2:**
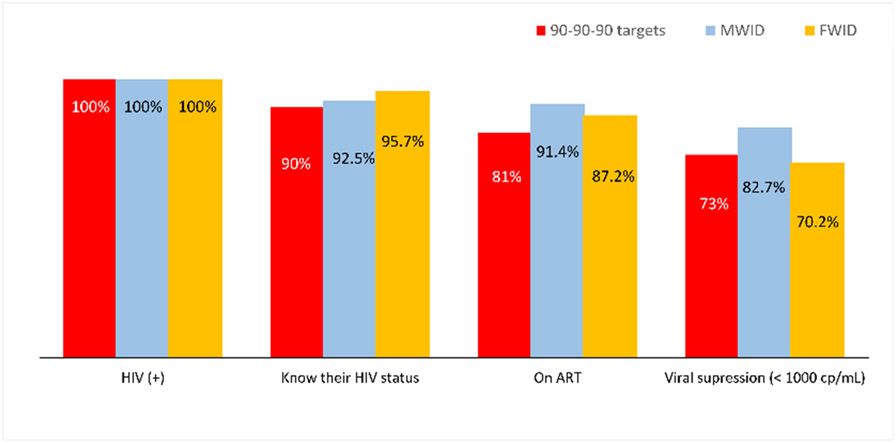
HIV cascade of care on the background of 90-90-90 targets by gender

The proportion of FWID who had methadone detected in urine was significantly lower than MWID (36.8% vs. 51.3%, respectively, Chi-square = 10.46, p < 0.01) (Table 1). In terms of the HIV cascade of care, several differences should be pointed out. While all the 90-90-90 targets were achieved in MWID, there was still a small gap in the third ‘90 goal’ for FWID; almost 30% who were HIV seropositive had an uncontrolled VL (threshold of 1000 copies/mL) while this proportion was of 17% for MWID (Fig. 2). Contact with peer-groups did not differ by gender (Table 1).

### Multivariable Associations with Gender

In multivariable logistic regression (Table 2), being a FWID was independently associated with being unemployed (adjusted odds ratio (aOR) = 1.9, 95% CI 1.2–3.0, p < 0.001), being sex worker (aOR = 23.1, 95% CI 9.2–58.7, p < 0.001), having unstable housing (aOR = 2.6, 95%CI 1.2-6.0, p < 0.05), reporting drug use for less than 5 years (aOR = 1.6, 95%CI 1.1–2.7, p < 0.05), reporting higher rates of methamphetamine use (aOR = 1.8, 95%CI 1.1–3.1, p < 0.05), and having a partner who ever injected drugs (aOR = 34.1, 95%CI 16.2–71.9, p < 0.001). Negative associations were seen for those having a higher education level (aOR = 0.2, 95%CI 0.1–0.4, p < 0.001) and less access to methadone treatment (aOR = 0.5, 95%CI 0.3–0.8, p < 0.01). The Mc Fadden’s Adj R^2^ indicator for the final multivariable model was 0.315.

## Discussion

To our knowledge, this is the first study examining differences in gender among PWID in a high HIV and HCV burden setting in Asia. Hai Phong has experienced a high seroprevalence HIV epidemic among PWID that began in the 1990s with an estimated 4,000–6,000 PWID [25].

FWID in our study had clear differences compared to men, which could potentially increase risk for health damage and HIV/hepatitis C transmission [26-32]. FWID often had lower levels of education, were more engaged in sex work, had greater difficulties finding work in the formal sector, and were more likely to have an unstable income and accommodations. Homelessness and unstable housing are strongly associated with an increased risk of HIV and HCV acquisition among PWID [33, 34]. Drug use and prostitution can become a vicious cycle in women’s lives; while stimulants are often used to get more customers and to achieve higher incomes [35, 36], buying stimulants also requires having more clients. As a result, FWID who sell sex often face double stigma. The dangers to women may sometimes come from their own partners; in our study, more than one third of FWID had a primary sexual partner who used drugs, and women had a higher risk of depression/anxiety and thoughts of harming oneself. FWID thus may have more difficulties normalizing their lives. These results are similar to studies of Manya Magnus et al. [26], Sabri et al. [27].

There was no difference in HIV and HCV infection prevalence among FWID and MWID. However, the lack of a difference in prevalence does not mean incidence is the same for each gender, as incidence might not be captured through the RDSS. Moreover, in this case-control comparison, the proportion of our study subjects reporting less than 5 years of injection drug use was higher among FWID compared to MWID, but the risk of HIV/HCV infection typically increases gradually over time for those reporting injection drug use, which has been shown in many previous studies [34, 37, 38]. In Hai Phong, HIV intervention programs implemented for the last 20 years have significantly controlled the spread of the disease, and the data from our cohort study clearly demonstrate that it is possible to achieve an end to an HIV epidemic among PWID in Hai Phong [39]. Nevertheless, there are differences on the third gap in the HIV cascade of care between FWID and MWID, which might be related to adherence, as access to ART is widely available and identification is typically not required. In contrast, HCV prevalence among PWID in Hai Phong remains extremely high and there is a lack of intervention strategies in the country as a whole [21]. In addition, the lower percentage of FWID with methadone positive urine compared to men points to inequalities in access to prevention services. With a sexist society like Vietnam, FWID, especially female sex workers, experience multiple forms of discrimination. They are often immigrants from other provinces, must stay in brothels, have no means of transportation and are controlled by their procurer. Meanwhile, the methadone treatment program, which is the only MOUD treatment available in Hai Phong, is quite strict in terms of administrative procedures, requiring support from relatives and daily medication [38]. Intersecting stigmas against PWID, especially FWID in the country, from all 3 sources of family, community, and even health workers, might contribute to poor access to methadone treatment in FWID, which has been shown in several recent studies in Vietnam [40, 41]. Therefore, closer monitoring of ART, increased access to methadone treatment (take home methadone), implementing buprenorphine treatment and decreasing methamphetamine use should be considered in supporting this hard-to-reach population.

Our results echo those of other studies, with some differences: in a convenience sample of younger PWIDs, Doherty et al. found women at higher risk of HIV infection but women were not more likely to be injected by their sexual partner when initiating injection [28]. Evans et al. found no significant differences between MWID and FWID in terms of education level, race, and housing [42].

This study shows that FWID have different needs, risk factors, and behaviors compared to MWID. Future studies are needed to develop improved prevention strategies for this population. High rates of sex partners who have ever injected drugs, depressive/anxiety symptoms and methamphetamine use among FWID highlight the need for gender-specific prevention approaches to slow HIV transmission. As mentioned in several studies, FWID can be a bridge for HIV transmission to the general population via sex risk behaviors [43, 44]. By focusing on injecting and HIV-related sexual behavior, this study complements the gender disparities data by socio characterizing differences, psychological issues and access to treatment services. Future studies to evaluate PWID continue to be needed to extend our knowledge of this population more fully.

There were several limitations in this study. As with most studies on risk behaviors for drug use and sexual behaviors, much of the information is gathered through self-report and participants may have difficulty recalling information or there might be social desirability bias in reporting undesirable behaviors such as sex work or unsafe sex. Of note, the partner CBOs dedicated to support workers in DRIVE reported that sex work was underreported. As an interviewer-administered questionnaire, there may be differences with respect to the interviewer reading the questions or recording the responses, despite extensive training. Continuous quality control had been undertaken to avoid these errors. Finally, as this was a cross-sectional study, we cannot establish temporality with respect to the results reported.

## Conclusion

In the Vietnamese context, FWID have less access to MOUD including methadone, they experience limited effectiveness of ARV treatment, are at higher risk for methamphetamine use and sexual risk, and have more need for mental health care compared to MWID. Interventions targeting this vulnerable population are needed, possibly through community organizations and peer educators.

## Figures and Tables

**Table 1:** Demographic, drug use, psychosocial characteristics and HIV and HCV serostatus by gender

	Male n = 620	Female n = 155	Statistical test value (Chi- Square or T-test test)	P value
**Demographic characteristics**				
Age (years) (mean ± SD)	36.8 ± 7.1 [36.2–37.4]	36.3 ± 7.2 [35.1–38.4]	-	
High school graduate and more	166 (26.8)	14 (9.1)	21.89	< 0.001
Having ID card	371 (59.8)	86 (55.5)	0.97	0.324
Having insurance health card	234 [37.7]	61 [39.4]	0.14	0.711
Total monthly income ≥ $250	253 (40.8)	78 (50.3)	4.59	0.032
Unemployed	194 (31.3)	79 (51.3)^a^	21.04	< 0.001
Sex worker	9 (1.5)	36 (23.2)	107.49	< 0.001
Unstable housing last 6 months	23 (3.7)	15 (9.7)	9.47	0.002
**Drug use behaviors**				
Heroin injecting time < 5 years (primarily injected drug in Vietnam)	162 (26.1)	59 (38.1)	8.67	0.003
Number of injections per month	26.9 ± 6.3	27.3 ± 6.4	−0.57*	0.568
Number of injections per day	2.2 ± 0.9	2.5 ± 1.0	−3.22*	< 0.001
Multi - drug use	452 (72.9)	123 (79.4)	2.7	0.101
***Non-injection drug use, last 6 months:***				
Methamphetamine	440 (71.0)	121 (78.1)	3.12	0.077
Cannabis	73 (11.8)	22 (14.2)	0.67	0.411
Ketamine	28 (4.5)	9 (5.8)	0.45	0.500
Ecstasy	25 (4.0)	10 (6.5)	1.68	0.194
Amphetamine	18 (2.9)	8 (5.2)	1.95	0.162
Cocaine	6 (1.0)	3 (1.9)	1.01	0.314
Methamphetamine positive in urine	225 (36.3)	71 (45.8)	4.76	0.029
Ever overdosed to the point of unconsciousness	27 (4.4)	10 (6.5)	1.2	0.273
**Psychosocial characteristics**				
Depressive/anxiety symptoms within the last 2 weeks (PHQ4 scale, total score ≥ 6)	52 (8.4)	18 (11.6)	1.57	0.210
Thought of harming oneself	60 (9.7)	27 (17.4)	7.46	0.006
**Risky practices**				
Unsafe sex activity	75 (12.1)	44 (28.4)	25.32	< 0.001
Partner ever injected drugs	14 (2.3)	59 (38.1)	186.33	< 0.001
Sharing needles/syringes/water	105 (16.9)	27 (17.4)	0.02	0.886
HIV seroprevalence	174 (28.1)	47 (30.3)	0.31	0.577
VL < 1000 copies/mL	140 (81.9)^b^	33 (71.7)^c^	2.30	0.129
HIV seroprevalence	433 (69.8)	104 (67.1)	0.44	0.508
Methadone detected in urine	318 (51.3)	57 (36.8)	10.46	0.001
Contact with peer-group	62 (10.0)	21 (13.6)	1.63	0.201

* T- test

a one value missing

b three viral load result missing

c one viral load result missing

**Table 2:** Logistic models of factors associated with being female among PWID in Haiphong, Vietnam (*N* = 775)

	Adjusted factors associated with being female aOR, [95%CI]^a^	P value
**Demographic characteristics**		
High school graduate and more	0.2 [0.1–0.4]	< 0.001
Total income ≥ 6.000.000	1.2 [0.7–1.9]	0.529
Unemployed	1.9 [1.2–3.0]	0.008
Sex worker	23.1 [9.2–57.8]	< 0.001
Unstable housing last 6 months	2.6 [1.2–6.0]	0.020
**Drug use behaviors**		
Heroin injecting time < 5 years	1.6 [1.1–2.7]	0.050
Multi - drug use	0.8 [0.4–1.4]	0.449
Methamphetamine positive in urine	1.8 [1.1–3.1]	0.021
**Psychosocial characteristics**		
Thought of harming oneself	1.4 [0.7–3.0]	0.334
**Risky practices**		
Unsafe sex activity	0.9 [0.5–1.7]	0.715
Partner ever injected drugs	34.1 [16.2–71.9]	< 0.001
Methadone detected in urine	0.5 [0.3–0.8]	0.008

a Mc Fadden’s Adj R^2^ = 0.315;

## Data Availability

The full data set contains highly sensitive information such as HIV serostatus and should not be made publicly available. The data can be made available through Data Use Agreements between a requester’s institution and the Hai Phong University of Medicine and Pharmacy. Interested persons should contact the authors at Hai Phong University of Medicine and Pharmacy.
